# The proteome of osteoblasts in a 3D culture perfusion bioreactor model compared with static conditions

**DOI:** 10.1038/s41598-025-96632-0

**Published:** 2025-04-09

**Authors:** Sonya Radi, Mostafa EzEldeen, Ákos Végvári, Dawn Coates, Reinhilde Jacobs, Nagihan Bostanci, Kai Bao

**Affiliations:** 1 OMFS-IMPATH Research Group, Faculty of Medicine, Department of Imaging and Pathology, KU Leuven and Oral and Maxillofacial Surgery, University Hospitals Leuven, Leuven, Belgium; 2 Department of Oral Health Sciences, KU Leuven and Paediatric Dentistry and Special Dental Care, University Hospitals Leuven, Leuven, Belgium; 3 Proteomics Biomedicum, Division of Chemistry I, Department of Medical Biochemistry and Biophysics (MBB), Karolinska Institutet, Solna, Stockholm, Sweden; 4 Sir John Walsh Research Institute, Faculty of Dentistry, University of Otago, Dunedin, New Zealand; 5 Department of Dental Medicine (DENTMED), Karolinska Institutet, Huddinge, Stockholm, Sweden; 6 Division of Oral Health and Periodontology, Department of Dental Medicine, Karolinska Institutet, Huddinge, Stockholm, Sweden

**Keywords:** Bone, Tissue engineering, Regenerative medicine, Human fetal osteoblastic cells, Proteomics, Bioreactor, Experimental models of disease, Bone

## Abstract

Bone disorders represent a significant global burden. Currently, animal models are used to develop and screen novel treatments. However, interspecies variations and ethical concerns highlight the need for a more complex 3D bone model. In this study, we developed a simplified in vitro bone-like model using a U-CUP perfusion-based bioreactor system, designed to provide continuous nutrient flow and mechanostimulation through 3D cultures. An immortalized human fetal osteoblastic cell line was seeded on collagen scaffolds and cultured for 21 days in both a perfusion bioreactor system and in static cultures. PrestoBlue™ assay, scanning electron microscopy, and proteomics allowed monitoring of metabolic activity and compared morphological and proteome differences between both conditions. Results indicated an altered cellular morphology in the bioreactor compared to the static cultures and identified a total of 3494 proteins. Of these, 105 proteins exhibited significant upregulation in the static culture, while 86 proteins displayed significant downregulation. Enrichment analyses of these proteins revealed ten significant pathways including epithelial-mesenchymal transition, TNF-alpha signaling via NF-kB, and KRAS pathway. The current data indicated of osteogenic differentiation enhancement within the bioreactor on day 21 compared to static cultures. In conclusion, the U-CUP perfusion bioreactor is beneficial for facilitating osteogenic differentiation in 3D cultures.

## Introduction

According to the World Health Organization (WHO), bone and joint diseases are the fourth most prevalent global health issue, underscoring the need to understand their pathophysiology and improve their treatments^1^. One example is osteoporosis, which affects approximately 10.2% of adults over the age of 50, is expected to become more prevalent in the coming years^2^. Fractures resulting from osteoporosis significantly impact a patients’ quality of life and can be challenging to treat. A common treatment for osteoporosis involves anti-resorptive medication, but this can lead to an adverse event known as medication-related osteonecrosis of the jaw, which affects the alveolar bone^3^. Currently, animal models are the golden standard for developing and screening novel treatments for bone-related pathologies. However, these models present several challenges, including ethical concerns, high costs, and need to induce human diseases which may not truly represent the human condition. Furthermore, bone density and turnover differ markedly between animals and humans^4^.

Tissue engineering and regenerative medicine (TERM) offer promising alternatives by mitigating some of the limitations posed by animal models and bridge the gap between clinical and translational research. A key aspect of TERM is the selection of an appropriate tissue engineering environment. While static cultures are widely used, they have limitations such as inefficient cell distribution and poor tissue development. The lack of circulation results in a model of diffusion-based nutrient transport, which limits uniform nutrient distribution^5,6^. Bioreactors overcome these limitations by providing a circulating environment that delivers nutrients and oxygen to the cells, while removing waste products, thereby promoting more uniform tissue development^5–7^. Additionally, bioreactors allow researchers to control and monitor environmental factors such as the perfusion rate, pH, temperature, and compound supplementation. Various bioreactor systems have been used in bone tissue engineering including spinner flask bioreactors, rotating bioreactors and electromagnetic field-based bioreactor systems. However, these systems have limitations such as unevenly distributed shear stress, poor mineralization effects in outer scaffold regions, and high costs^8^. Perfusion-based bioreactors emerged as the most suitable for our specific application in bone tissue engineering due to the artificial mechanostimulation and the unique perfusion system they provide^9,10^. Previous studies have shown that providing mechanical stimulation promotes the development of bone-like structures through various mechanoreceptors and by mimicking muscle forces^9,11–14^. This stimulation is particularly beneficial in activating Piezo channels in bone cells, where mechanical stress induces ion flux, enhancing cellular mechanostimulation^9^. In osteoblasts, Piezo1 channels are responsible for migration while in osteoclasts, it is responsible for the indirect regulation of bone resorption^15^. In this research, another key motivation for using perfusion-based bioreactors is their compatibility with standard cell culture incubators, making them easily accessible for any laboratory. Besides, the U-shaped bioreactor has been shown to enhance cell seeding efficiency and homogeneous cell distribution^16,17^.

We used a proteomic-based method to assess the effect of bioreactor perfusion on osteogenic differentiation and to provide an overall proteomic profile. This approach allows for large-scale high-throughput comparative analyses that offers deep insights into mechanistic pathways and key protein regulators^18^. Compared to studies using transcriptomics, proteomics provides a more accurate representation of the final products of cellular processes^19^. In this study hFOB1.19 were used due to their osteogenic capabilities and commercial availability which helps standardizing this model^20,21^. Additionally, Collagen scaffolds were used as collagen is a major component of the bone extracellular matrix (ECM) and has beneficial characteristics such as biocompatibility, low antigenicity, and ability to support the adhesion, proliferation, and differentiation of bone cells^22,23^. The high porosity of these scaffolds also promotes uniform nutrition distribution, which is critical when using a perfusion bioreactor system.

Animal models, though commonly used for such research, have significant limitations and fail to model human biology. We hypothesized that a dynamic perfusion bioreactor would provide an environment that enhances osteogenic differentiation by targeting the involved pathways. Using the commercialized fetal osteoblastic cell line, this standardized model could serve as a globally accessible tool for bone research. Additionally, these cells are pre-programmed for osteoblastic differentiation and do not require added supplements or modifications to the culture environment to induce osteogenic differentiation^24^. Therefore, this research focuses solely on the effects of perfusion in the U-CUP bioreactor system on osteogenic differentiation. This study aimed to develop a bioreactor model for bone tissue engineering while investigating the molecular basis of this model. To our knowledge, this is the first study to explore the proteomic basis of osteogenic differentiation within the U-CUP bioreactor system.

## Results

### Osteoblast metabolic activity and the morphological status in static and bioreactor models

**Fig. 1:**
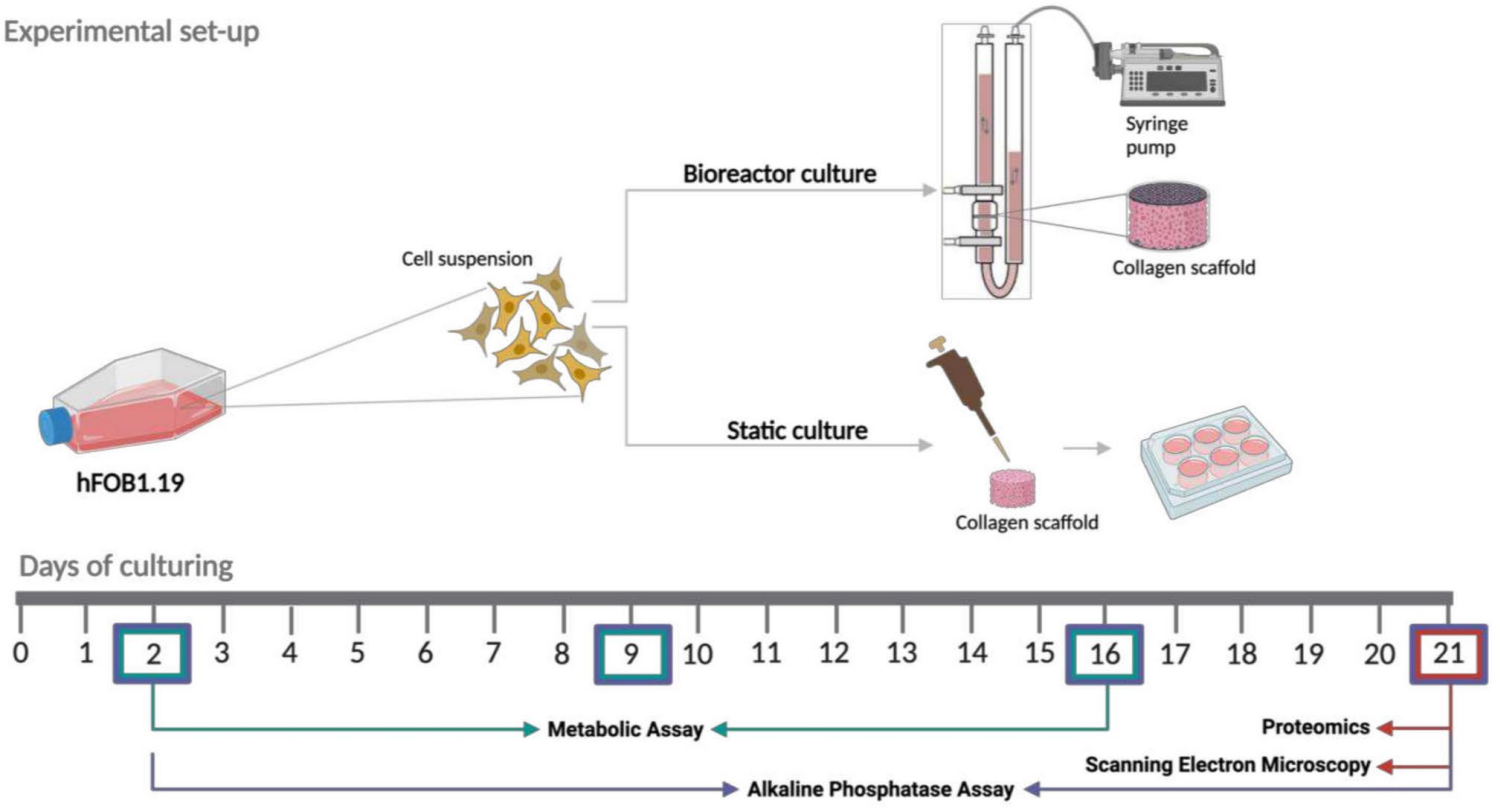
An overview of the experimental set-up of the bioreactor culture and static culture. A schematic illustration of the set-up and timeline of the experiments. This figure was created with BioRender.com.

**Fig. 2:**
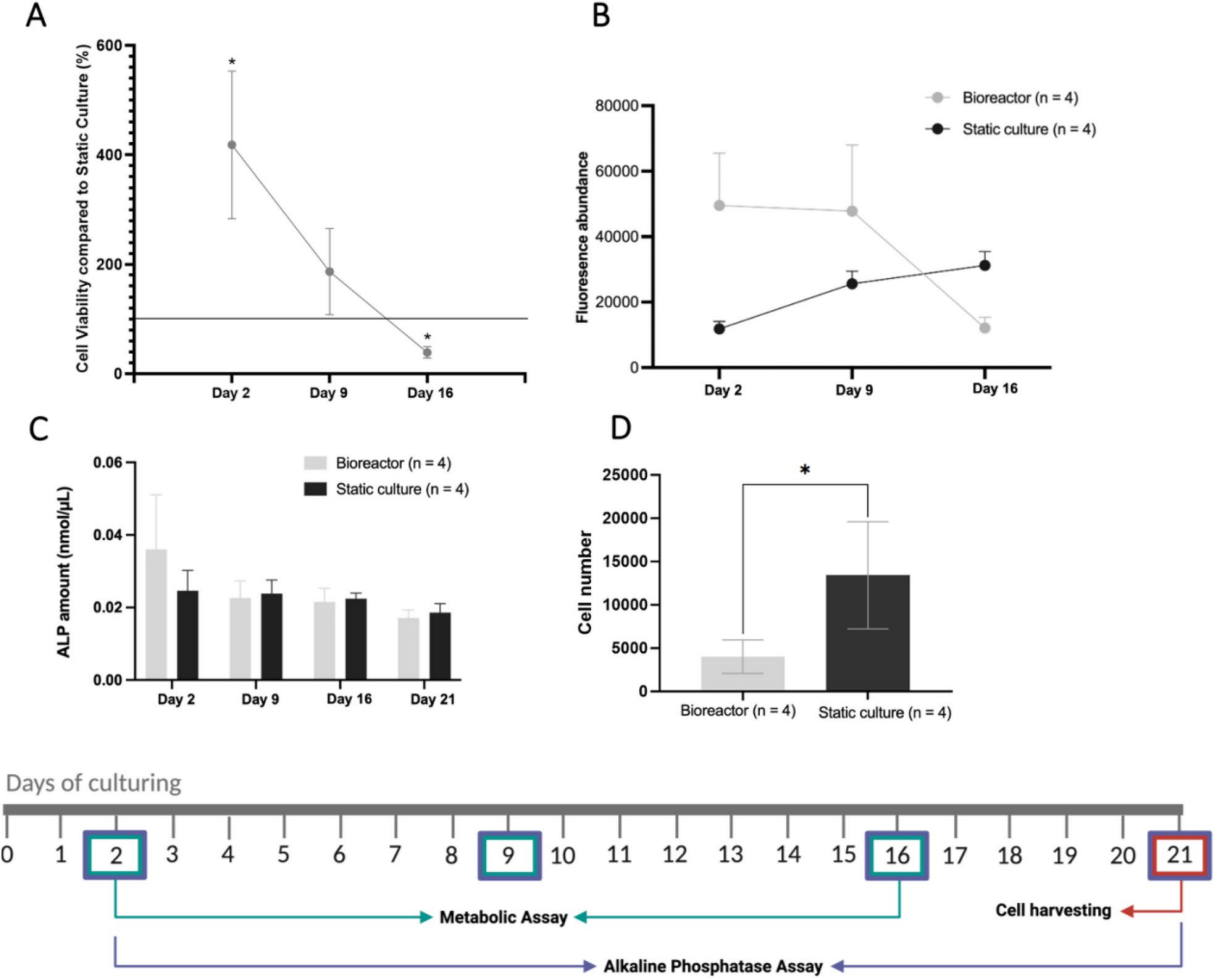
The results of the PrestoBlue™ metabolic assay, Alkaline phosphatase assay, and cell dissociation results of the bioreactor (n = 4) compared to the static cultures (n = 4). (**A**) PrestoBlue™ metabolic Assay results. The relative cell viability of bioreactor compared to static cultures are shown. (**B**) PrestoBlue™ metabolic Assay results. The fluorescence abundance of bioreactor and static cultures are shown. (**C**) Alkaline Phosphatase Assay results. The mean ALP amount on days 2, 9, 16, and 21 of the bioreactor compared to static culture is shown. (**D**) Cell Dissociation results. Cell counting results of the bioreactor and static culture on day 21. For all values, the mean and standard deviations are shown. ALP = Alkaline phosphatase, *P value < 0.05.

**Fig. 3:**
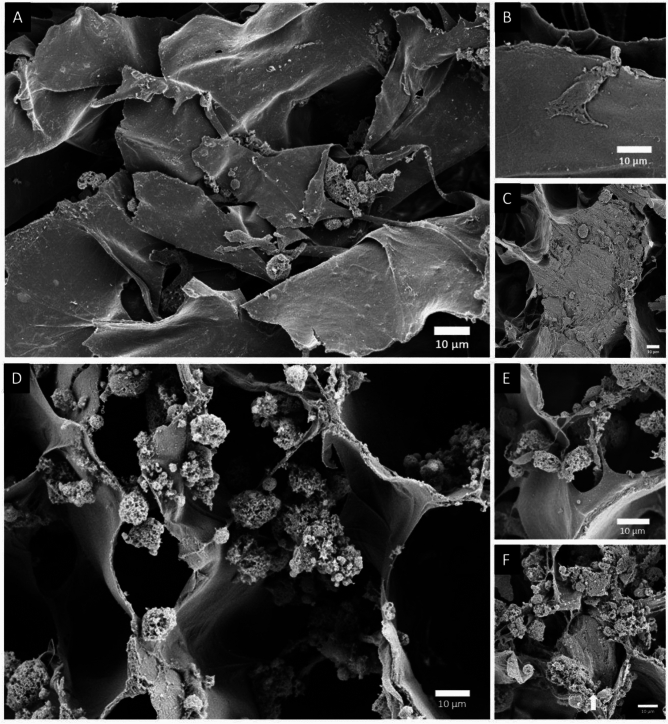
Scanning Electron Microscopy micrographs of the collagen scaffold with hFOB1.19 cells grown for 21 days in a dynamic perfusion bioreactor system or in a static system (**A**) An overview of the collagen scaffold in the bioreactor with a magnification of 500×. (Scale bar set at 10 µm) (**B**) A hFOB1.19 cell in the bioreactor shown with a magnification of 500×. (Scale bar set at 10 µm) (**C**) hFOB1.19 cells embedded in the collagen scaffold shown in the bioreactor with a magnification of 500×. (Scale bar set at 10 µm) (**D**) An overview of the collagen scaffold in static conditions with a magnification of 500×. (Scale bar set at 10 µm) (**E**) hFOB1.19 cells shown in static conditions with a magnification of 500×. (Scale bar set at 10 µm) (**F**) hFOB1.19 cells embedded in the collagen scaffold shown in static conditions with a magnification of 500×. (Scale bar set at 10 µm).

To evaluate the impact of a dynamic environment on the metabolic activity and indirectly the viability of the hFOB1.19 cells in perfusion bioreactor and static cultures, a PrestoBlue assay was performed on days 2, 9, and 16 (Figs. 1, 2A,B). On day 2, the bioreactor cultures showed significantly higher relative cell metabolic activity compared to the static cultures (ratio: 4.18:1, *P* value = 0.035). However, by day 16, the relative metabolic activity in the bioreactor cultures was significantly lower (ratio: 0.39:1, *P* value = 0.002). On day 21, the mean cell number from the bioreactor culture (4000) was significantly (*P* value = 0.027) lower than the mean cell number from the static culture (13 406). The osteoblastic marker alkaline phosphatase (ALP) was quantified in both conditions over the 21-days as a measure of osteogenic differentiation. However, its levels remained constant over time, with no significant differences observed between the groups (Figs. 1, 2C). To analyze the scaffold morphology and assess cell attachment, the samples were examined using SEM. Scaffolds retrieved from the dynamic perfusion bioreactor (Fig. 3A–C) showed fewer cells, which is also supported by the results of cell counting (Fig. 2D, Supplementary table 1) and live/dead staining (Supplementary Fig. 1) on day 21. These cells have a more spread-out and smoothened morphology compared to the more round and scattered morphology in scaffolds from static cultures (Fig. 3D–F).

### Proteomic analysis of the differentiation of hFOB1.19 cells in a dynamic VS static bone tissue model

**Fig. 4:**
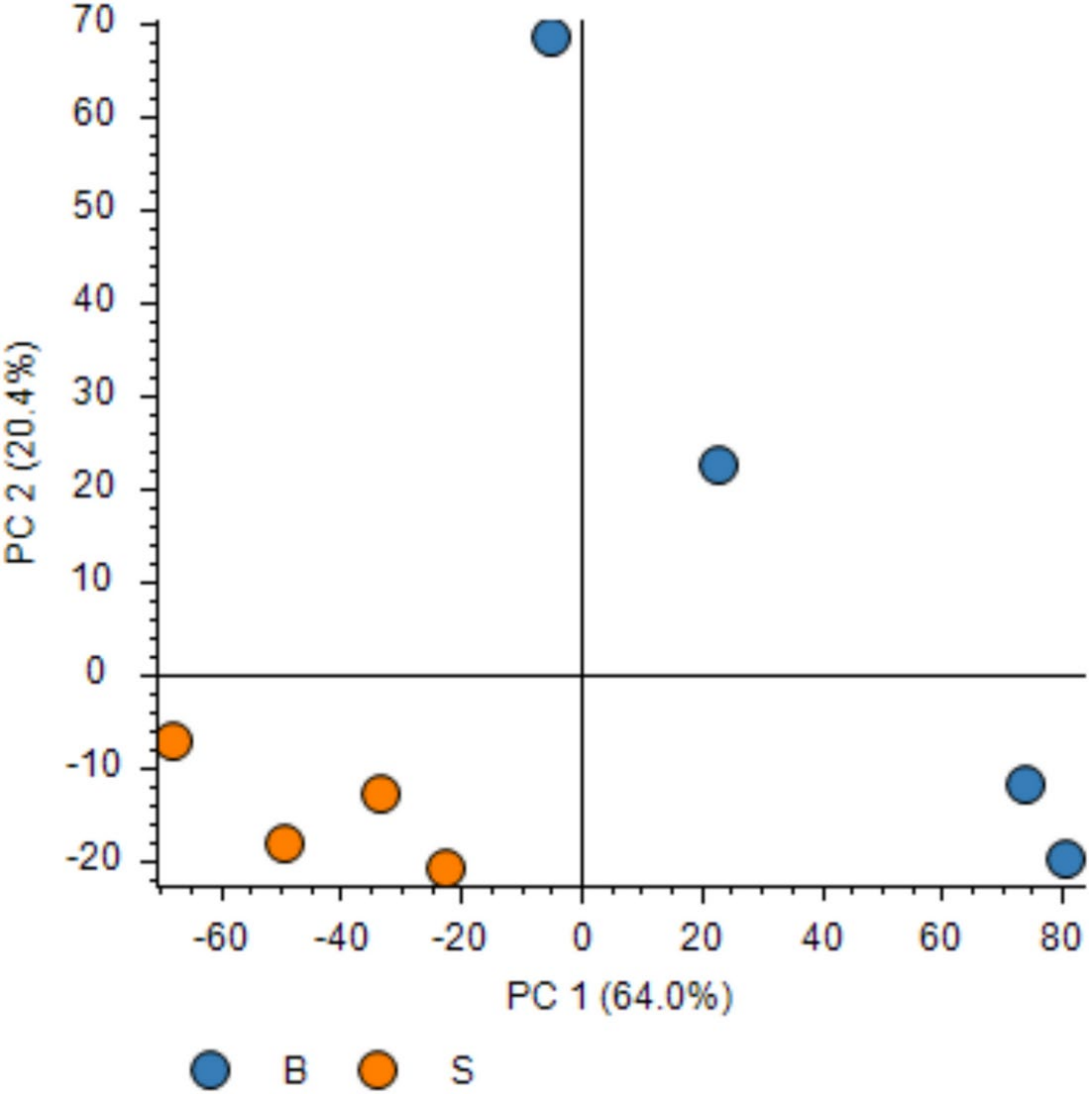
Principle component analysis (PCA) showing sample grouping based on protein abundances of 1897 quantified proteins. PC1 explained 64.0% of the variants while PC2 explained 20.4%. Bioreactor (blue dots) and static cultures (orange dots).

To provide a comprehensive picture of how bioreactors impact the differentiation potential of hFOB1.19, the proteomic changes were analyzed in both bioreactor (n = 4) and static cultures (n = 4). A total of 3494 proteins were identified (Supplementary Table 2). From these identified proteins, 3466 were shared among all samples, with 27 unique to the bioreactor and 1 protein unique to static cultures, respectively. Furthermore, principal component analysis (PCA) plot of proteome profiles projected onto the first two principal components (PC1 and PC2) demonstrated a clear separation between bioreactor and static culture samples. The bioreactor samples (blue) are positioned on the right, while the static culture samples (orange) are positioned on the left, reflecting separation based on PC1 (Fig. 4).

### Functional enrichment analysis of differentially abundant proteins

Among the 3466 overlapping proteins, 105 exhibited significant upregulation (Abundance Ratio: (S)/(B) > 0 *p* adj < 0.05) in static cultures compared to bioreactor cultures, while 86 proteins displayed significant downregulation (Abundance Ratio: (S)/(B) < 0 *p* adj < 0.05) (Supplementary Table 3). The top ten abundant proteins in bioreactor and static cultures are shown in Table 1. Among them, proteins involved in DNA replication and fibrinolysis, such as HMGA1 and PAI2, were shown to be abundant in bioreactor cultures. In the static cultures proteins associated with apoptosis, including S100A8, HSP76, and LEG7, were abundant.

**Table 1:** Top ten abundant proteins in bioreactor and static cultures.

Accession	Description	Log_2_ (abundance S/abundance B)	Abundance ratio Adj. * P* value: (S)/(B)
Static cultures			
P05109	Protein S100-A8	− 4.84	7.14 × 10^–14^
P17066	Heat shock 70 kDa protein 6	− 4.44	5.98 × 10^–15^
P04259	Keratin, type II cytoskeletal 6B	− 3.52	2.31 × 10^–10^
P42357	Histidine ammonia-lyase	− 3.51	1.45 × 10^–8^
O00743	Serine/threonine-protein phosphatase 6 catalytic subunit	− 3.51	4.31 × 10^–7^
Q08554	Desmocollin-1	− 3.28	1.65 × 10^–6^
P47929	Galectin-7	− 3.07	2.67 × 10^–7^
P13196	5-aminolevulinate synthase, non-specific, mitochondrial	− 2.97	1.33 × 10^–6^
P04264	Keratin, type II cytoskeletal 1	− 2.83	1.51 × 10^–7^
P02511	Alpha-crystallin B chain	− 2.68	1.23 × 10^–7^
Bioreactor cultures			
P0DME0	Protein SETSIP	4.12	2.3 × 10^–5^
P82979	SAP domain-containing ribonucleoprotein	4.14	8.7 × 10^–7^
Q8NC51	Plasminogen activator inhibitor 1 RNA-binding protein	4.15	3.72 × 10^–5^
Q9Y3U8	60S ribosomal protein L36	4.19	1.02 × 10^–5^
P17096	High mobility group protein HMG-I/HMG-Y	4.23	1.65 × 10^–5^
P01584	Interleukin-1 beta	4.38	1.17 × 10^–6^
P05120	Plasminogen activator inhibitor 2	5.00	1.13 × 10^–8^
P16401	Histone H1.5	5.00	9.18 × 10^–9^
P06454	Prothymosin alpha	5.00	2.51 × 10^–8^
P16402	Histone H1.3	5.39	2.25 × 10^–10^

The abundances of the different proteins are shown together with the adjusted *P* value and their related biological process, molecular function, and cellular component.

To elucidate the functional roles of these proteins over-representation analysis (ORA) utilizing the Hallmark database (Table 2, Supplementary Table 3, Supplementary Table 4). The analysis of the ORA identified “Epithelial mesenchymal transition (EMT)”, “TNFA signaling via NF_B”, and “KRAS signaling” as the most significantly regulated pathways with an FDR less than 0.002. Respectively, 19, 11, and 15 proteins were identified in these pathways (Supplementary Table 4). Intriguingly, all three pathways have been previously reported to be associated with osteogenic differentiation or mineralization.

**Table 2:** Results of the ORA analysis of the proteins of the bioreactor cultures and static cultures.

Description	Regulated protein/all protein	FDR
Epithelial mesenchymal transition	19/86	0.0002
TNFA signaling via NF_B	11/36	0.0006
KRAS signaling, downregulated genes	6/12	0.0014
KRAS signaling, upregulated genes	9/29	0.0015
Complement cascade	12/60	0.0088
UV response: upregulated genes	12/64	0.0134
Response to hypoxia; HIF1A targets	13/75	0.0155
Inflammation	7/30	0.0283
Glycolysis and gluconeogenesis	13/93	0.0801
Biosynthesis of bile acids	6/30	0.0920

*FDR* false discovery rate.

**Fig. 5:**
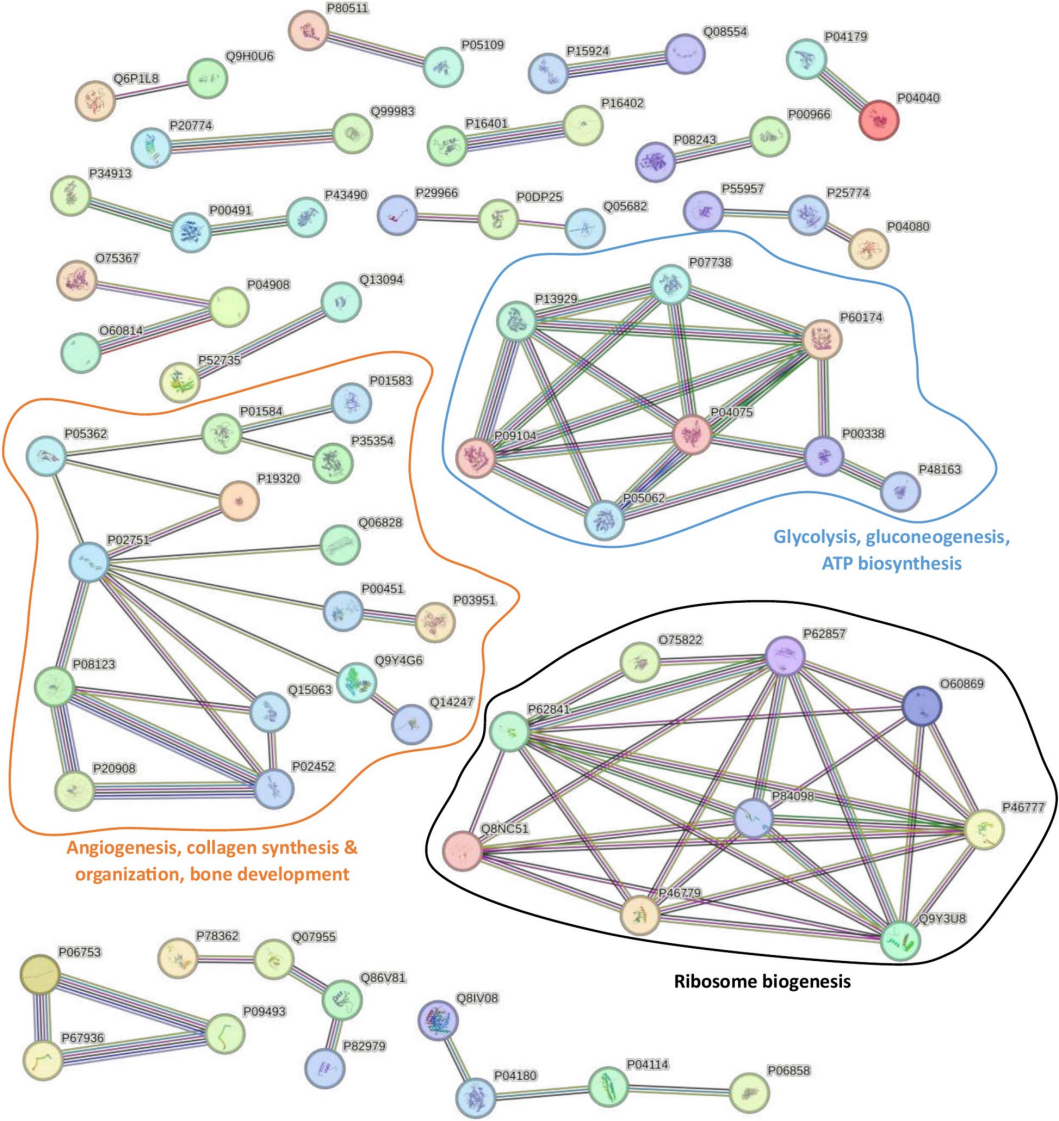
Protein–protein interaction analysis and overrepresentation enrichment analysis of the significant proteins. The String online tool was used to gain insights into the interactions and functions of these proteins. Each node is representative of a protein. Red node = extracellular region, purple node = extracellular space, green node = secreted, white node = second shell of interactors, filled node = 3D structure is known or predicted, empty node = unknown 3D structures. Each line represents protein–protein interactions. Light blue and pink interactions refer to known interactions (from databases or experiments). Green, red, and dark blue interactions refer to predicted interactions (gene neighborhood, fusions, and co-occurrence). Yellow line = text mining, black line = co-expression, light purple line = protein homology.

To further understand the interaction between the regulated proteins, a protein–protein interaction (PPI) analysis was performed using the String online tool (https://string-db.org), which integrates both known and predicted PPIs (Fig. 5, Supplementary Table 5). Active interaction sources and an interaction score > 0.9 were applied to construct the PPI networks. The PPI networks identified few clusters of highly interconnected nodes. The multiple interactions of those proteins enriched in the selected pathways were represented by different color lines. The results showed that 115 of these regulated proteins were from the extracellular region, 110 were from the extracellular space, and 27 were from the ECM which suggests that our bioreactor possibly impacted the ECM (Supplementary Table 6). Three main clusters were identified involved in different biological processes (Fig. 5). The largest cluster involved PPI related to angiogenesis, bone formation, cell adhesion, and motility. The remaining two clusters involved PPI related to protein synthesis and energy metabolism, respectively. Overall, these proteomics results suggest that the bioreactor culture affects the hFOB1.19 cell differentiation by modulating the expression of specific proteins and pathways involved in cell motility and ECM regulation.

## Discussion

In this study, we developed a 3D culture model using hFOB1.19 cells in static and perfusion bioreactor conditions and conducted a comprehensive quantitative proteome analysis. While static cultures are commonly used, they rely heavily on diffusion for nutrient and oxygen delivery, which can negatively impact cell metabolic activity, and indirectly viability over time^25^. The perfusion provided by the bioreactor was hypothesized to mimic the dynamic bone microenvironment, potentially promoting osteogenic differentiation^5,9,10^. Our results align with previous findings indicating that osteogenic differentiation requires approximately three weeks^26^. Consistent with the hFOB1.19 cell line’s characteristics, cell metabolic activity decreased over time in the bioreactor culture compared to the static cultures likely due to an increase in differentiation^21^. These results suggested that bioreactor cultures provide better nutrient access, although prolonged culturing leads to a reduction in cell metabolic activity. Among the bioreactor cultures, variability was higher in the earlier stages but stabilized over time, while metabolic activity declined after day 9. Importantly, the ALP content remained unchanged, highlighting day 9 as a crucial time point for differentiation. In contrast, the static culture exhibits an increase in metabolic activity, suggesting that in this condition the cells were primarily proliferating rather than differentiating.

SEM imaging qualitative analysis revealed that within the static cultures, cells exhibited a round scattered morphology and were more connected to the scaffold by cell microfilaments. In contrast, cells within the bioreactor cultures exhibited a smoothened and spread-out morphology^27,28^. Fewer cells are visible in the dynamic bioreactor culture, which could be attributed to osteogenic differentiation as apoptosis is one of the terminal outcomes for osteoblasts^29^. Furthermore, the involved mechanisms of the bioreactor culture were further elucidated by the top four significantly enriched pathways, as identified through ORA analysis. The most significantly regulated pathway was EMT, a process in which epithelial cells gain mesenchymal characteristics, lose their adhesion structures, and become motile^30^. In this pathway proteins such as transforming growth factor beta receptor type 3, important for the bone morphogenetic protein signaling pathway and cell migration, were identified^31,32^. To form bone, it is critical that precursor cells can move to be recruited and to form the correct bone shape^33^. Past research has shown that EMT promotes osteoblastic differentiation^34^. Additionally, EMT transcription factors such as ZEB1 and SNAIL can inhibit osteoblastic differentiation, so their downregulation supports differentiation^35^. In the bioreactor culture, the EMT pathway was downregulated on day 21. Since EMT supports precursor cell motility essential for bone formation, its downregulation at a later stage likely reflects osteogenic differentiation completion^33^. Two other significantly regulated pathways were TNF-alpha signaling via NFĸB, and KRAS signaling. TNF-alpha promotes osteogenic differentiation by activating the NFĸB pathway, which upregulates osteogenic markers^36^. Additionally, the NFĸB pathway increases TAZ expression, further enhancing osteogenic differentiation^37,38^. Past research has shown that the NFĸB pathway can stimulate key osteogenic regulators BMP2, RUNX2, and osterix, and promote mineralization^37^. While less frequently associated with osteogenic differentiation, the KRAS signaling pathway, involving the small GTPase KRAS, has been identified as an osteogenic regulator influencing ECM accumulation and mineralization ^39,40^.

Additionally, PPI analysis revealed three primary clusters with patterns among regulated proteins that further support our findings. The largest cluster included proteins associated with angiogenesis such as prostaglandin synthase, fibronectin, and various collagen chains. Proteins related to collagen synthesis, organization, and bone development were also identified, such as fibromodulin and periostin. Other proteins in this cluster were related to cell adhesion, cell motility, and cytoskeleton organization. These biological processes are critical for osteogenic differentiation. Angiogenesis, essential for bone formation by ensuring the supply of nutrients, oxygen, and cells, is stimulated by osteoblasts through their interaction with endothelial cells^41^. As mentioned, the perfusion in the bioreactor leads to a more uniform distribution of oxygen and nutrients. These results suggest that oxygen tension may be a key factor contributing to the enhancement of osteogenic differentiation. Collagen synthesis and organization are also crucial hallmarks of osteogenic differentiation, with collagen being a predominant protein in the bone ECM^42^. Osteoid, primarily produced by osteoblastic cells during bone formation, is an unmineralized ECM composed of mainly collagen type 1 and provides a nucleation point for mineralization^43^. The second-largest cluster involved ribosomal subunit proteins, reflecting the dynamic regulation of ribosome biogenesis during osteoblast differentiation^44^. The smallest cluster included proteins linked to glycolysis, gluconeogenesis, and ATP biosynthesis, highlighting the metabolic demands of osteogenic differentiation^45^.

When examining the most abundant proteins for both conditions, the bioreactor culture shows SETSIP as the most abundant protein. This protein plays a role in endothelial cell differentiation, which, as previously mentioned, is critical for the osteoblast-endothelial cell communication during angiogenesis. Other abundant proteins in the bioreactor culture are associated with protein synthesis and DNA replication, such as SARNP, SERB1, and HMGA1. DNA replication is essential for the epigenetic regulation, while protein synthesis is integral to cell differentiation, proliferation, and function. Another noteworthy protein in the bioreactor culture is PAI2 or plasminogen activator inhibitor 2, which primarily contributes to fibrinolysis—a process commonly associated with wound healing. However, fibrinolysis also plays a role in bone development and osteogenic differentiation^46,47^. Following a fracture, fibrinolysis is necessary for breaking down the fibrin clot to aid bone healing. In contrast, the static culture exhibits a different set of abundant proteins. The most abundant protein, S100A8, is a calcium- and zinc-binding protein which has a wide plethora of intra- and extracellular functions. The extracellular functions involve pro-inflammatory, antimicrobial, oxidant-scavenging, and apoptosis-inducing activities^48,49^. Increased apoptosis is linked to reduced osteogenic potential because the loss of osteoblasts decreases the rate of bone formation^50,51^. This is supported by the fact that, although the bioreactor culture contained fewer cells, it did not show a decrease in ALP activity. Therefore, these results suggest that osteogenic differentiation progressed more efficiently in the bioreactor culture and most likely resulted in apoptosis. The second most abundant protein, HSP76, is a heat shock protein produced by cells in stressful conditions. Research has shown that heat shock proteins can regulate the osteogenic differentiation of human adipose-derived stem cells. However, an overexpression of these proteins may suppress osteogenic differentiation, though this mechanism requires further investigation across different osteogenic precursor cell lines^52^. Surprisingly, no proteins directly associated with mechanostimulation were identified. However, the proteomic analysis revealed that nine of the regulated proteins are linked to integrin cell surface interactions, as evidenced by the related reactome pathways, highlighting the involvement of mechanical cues.

This study has some limitations that need to be recognized. There was no validation of possible mineralised structures. Future research should address this by incorporating techniques such as Raman spectroscopy, energy-dispersive X-ray spectroscopy, and X-ray diffraction analyses to quantify mineralization. This study also did not include calculations or direct measurements of shear stress, or the mechanical properties of the biomaterials involved. Besides, this study provides a snapshot of the involved cultures. In further research, it would be interesting to consider assessing the cell number and expression of osteogenic biomarkers over time. Performing an RT-qPCR would be a valuable tool to, for example, further establish the role of ALP in the bioreactor culture.

As for the implications of our findings, this study establishes a foundational model for advancing bone tissue constructs and research in bone regeneration and remodeling. The U-CUP bioreactor showed promising results by enhancing osteogenic differentiation through the representation of dynamic in vivo-like conditions, which provide improved oxygen and nutrient distribution, as well as mechanical stimulation. Proteomic analysis highlighted non-traditional pathways, such as EMT and KRAS signaling, and identified proteins like SARNP and SERB1 that warrant further exploration. While exploratory, these findings underscore the potential of the U-CUP bioreactor in bone tissue engineering.

## Methods

### Cell culture

To establish the 3D cell culture, immortalized human fetal osteoblastic cells (hFOB1.19, American Type Culture Collection, Virginia, USA) were used^53^. These cells were cultured in a 1:1 mixture of Dulbecco’s Modified Eagle Medium/Nutrient Mixture F-12 (DMEM/F-12, HEPES, Gibco, Thermo Fisher Scientific, USA) supplemented with 10% fetal bovine serum (FBS, Gibco, Thermo Fisher Scientific, USA), 1% penicillin/streptomycin (Gibco, Thermo Fisher Scientific, USA), and 0.3 mg/mL Geneticin (G418, Gibco, Thermo Fisher Scientific, USA).

### Preparation of the perfusion bioreactor and static cultures

The 3D culture was constructed using collagen sponges (porcine collagen type I, O3D304030, Optimaix, Matricel GmbH)^54^. Disc-shaped collagen scaffolds with a diameter of 8 mm and a thickness of 3 mm were cut using a disposable biopsy punch (8 mm, KAI medical, Japan). Within the bioreactor system, the scaffolds were embedded within a pair of adaptors inside the bioreactor chamber (UCUP, Cellec Biotek AG, www.cellecbiotek.com, UCUP001). Per scaffold, 1 × 10^6^ hFOB1.19 cells in growth medium were seeded at a velocity of 1000 µm/s overnight using the PHD Ultra Syringe Pump^55^. The day after, the velocity was changed to 100 µm/s (Fig. 1)^55^. Furthermore, it was ensured that there were no air pockets or bubbles around the scaffold. For the control groups, referred to as the static culture, the scaffolds were placed in 6-well culture plates and seeded with 1 × 10^6^ hFOB1.19 cells per construct. After 0.5 h, growth medium was added, and the day after, the constructs were moved to new plates and the medium was refreshed. Perfusion and static cultures were maintained for 21 days (37 °C, 5% CO_2_), with the medium changed every other day. The volume of the medium was the same for both conditions.

### PrestoBlue™ metabolic assay

On day 2, 9, and 16 a PrestoBlue™ metabolic assay was performed. Static cultures and bioreactor systems were incubated at 37 °C with 5% CO_2_ for 4 h with 10% (v/v) PrestoBlue™ reagent (A13262, Thermo Fisher Scientific, USA) and then 100 µL of the supernatant were harvested from each culture/system. Fluorescence was measured using a CLARIOstar® spectrophotometer (BMG Labtech, Germany) at Ex/Em 560-10/590-10 nm. Cell viability (%) was calculated by dividing the bioreactor’s fluorescence abundance by the static cultures’ fluorescence abundance. The mean value and standard deviation were calculated, the normality was checked, and Welch’s t test was performed using GraphPad.

### Alkaline phosphatase assay™

Medium derived from the cultured cell-loaded scaffolds was filtered and used for testing alkaline phosphatase using an Alkaline Phosphatase Assay Kit (Abcam, UK). The samples were diluted with Assay buffer provided with the kit and incubated for 30 min at room temperature with 50 mM 4-Methylumbelliferyl Phosphate (MUP) substrate. Samples diluted with Assay buffer and incubated with the stop solution provided with the kit were used to correct for the background signal. The fluorescence of the ALP assay was measured (Ex/Em 360/440 nm) using a CLARIOstar® spectrophotometer (BMG Labtech, Germany). The ALP amount was calculated per sample following the manufacturer’s protocol. The mean value and standard deviation were calculated, the normality was checked, and two-way ANOVA was performed using GraphPad.

### Scanning electron microscopy

The collected samples were washed with 1X DPBS (Gibco, Thermo Fisher Scientific, USA) and fixed using 2.5% glutaraldehyde in 0.1 M phosphate buffer, pH 7.4. The fixed samples were washed in MilliQ water prior to stepwise ethanol dehydration and critical-point-drying using carbon dioxide (Leica EM CPD 030). The samples were mounted on specimen pins using double sided carbon adhesive tabs and sputter coated with a 10 nm layer of platinum (Quorum Q150T ES). SEM images were acquired using an Ultra 55 field emission scanning electron microscope (Zeiss, Oberkochen, Germany) at 5 kV and the SE2 detector.

### LIVE/DEAD™ staining

The LIVE/DEAD™ Viability/Cytotoxicity Kit, for mammalian cells (Invitrogen, ThermoFisher Scientific, USA) was used. The live/dead solution (DPBS, 4 µM Ethidium Homodimer-1, 2 µM Calcein AM) was prepared. The scaffold pieces were incubated in 1 mL live/dead solution for 30 min (37 °C). Using a fluorescence microscope (Nikon, Japan) the fluorescence of the live/dead staining was visualized (Ethidium Homodimer-1: Ex/Em 528/617 nm, Calcein AM: Ex/Em 494/517 nm). The results were captured using the NIS elements F software (Nikon, Japan).

### Cell dissociation protocol

The collagen scaffold was washed three times with 1X DPBS (Gibco, Thermo Fisher Scientific, USA). Subsequently, the scaffold was cut into pieces and incubated three times with a trypsin/ethylenediaminetetraacetic acid solution (0.25%, Sigma-Aldrich, USA) for 5 min at 37 °C. After each incubation period, the scaffold was flushed 20 times vigorously using a P1000 pipette, and the suspension was saved in a falcon tube containing three volumes of complete DMEM/F-12. Later, the cell suspension was filtered using a cell strainer (70 µm Nylon, Corning®) to separate the cells from the scaffold debris. The filtered cell suspension was centrifuged at 0.3 g for 5 min. The cell pellet was then collected and placed at − 20 °C for further analyses.

### Protein digestion

The cell pellet obtained from perfusion (n = 4) and static cultures (n = 4) was utilized. Proteins were lysed, digested, and purified using the PreOmics SP3-iST kit (PreOmics GmbH, Germany) following the manufacturer’s protocol. The only adaptation was the last step of the protein lysis protocol where the samples were spun down, sonicated for 60 s with high-energy sound waves (amplitude 100%, CT, P 0–7 W, C 70%) and spun down again. These peptide extracts were then concentrated using a Speedvac (Thermo Savant SPD121P, Thermo Scientific, Waltham, MA, USA) and stored at − 20 °C until further use.

### Liquid chromatography-tandem mass spectrometry data acquisition

Peptides obtained from perfusion (n = 4) and static cultures (n = 4) were utilized. Peptides were prepared by reconstituting them in 20 µL of solvent A (2% acetonitrile, 0.1% formic acid) and injecting 3 µL onto a 50 cm long EASY-Spray C18 column connected to an Ultimate 3000 nanoUPLC system. This system used a 90-min gradient of solvent B (98% acetonitrile, 0.1% formic acid) ranging from 4 to 26% for 90 min, followed by 26–95% for 5 min, and 95% for 5 min at a flow rate of 300 nL/min. The mass spectra were obtained on a Q Exactive HF hybrid quadrupole Orbitrap mass spectrometer (Thermo Fisher Scientific) in the range of *m/z* 375–1800 and at a resolution of R = 120,000 (at *m/z* 200) targeting 5 × 10^6^ ions for a maximum injection time of 100 ms. This was followed by data-dependent higher-energy collisional dissociation (HCD) fragmentations of precursor ions with a charge state of 2 + to 8 +, using 45 s dynamic exclusion. The tandem mass spectra of the top 17 precursor ions were acquired with a resolution of R = 30,000, targeting 2 × 10^5^ ions for a maximum injection time of 54 ms, setting the quadrupole isolation width to 1.4 Th and the normalized collision energy to 28%.

### Protein quantification

The acquired raw data files were analyzed using Proteome Discoverer v3.0 (Thermo Fisher Scientific). The MS Amanda search engine was used to search against the human protein database (SwissProt taxon ID: 9606 with 20,359 entries, version 2024-03-07). The search parameters included allowing for a maximum of two missed cleavage sites for full tryptic digestion and setting the precursor and fragment ion mass tolerance to 10 ppm and 0.02 Da, respectively. The static modification of carbamidomethylation of cysteine and the dynamic modifications of acetylation on N-termini, oxidation of methionine, and deamidation of asparagine and glutamine were also specified. The initial search results were filtered using the Percolator node in Proteome Discoverer, with a 1% and 5% protein false discovery rate (FDR). Quantification was based on the precursor intensities of unique peptides, and the data are expressed as the mean ± standard deviation. Statistical testing was performed using Proteome Discoverer v3.0 (Thermo Fisher Scientific) and the significance was determined using P adjust value (p-adj) < 0.05 as significant.

### Bioinformatic analysis

We performed an over-representation enrichment analysis (ORA) using regulated proteins against all identified proteins as a background against on WebGestalt online tool (https https://www.webgestalt.org/, accessed on November 26th, 2024) against the Hallmark database. The top 10 enriched results based on FDR were reported. To gain further insights into the interactions between the significantly regulated proteins, we analyzed protein–protein interactions using the STRING database (Search Tool for the Retrieval of Interacting Genes/Proteins; http://string-db.org/, accessed on May 10, 2023). The protein–protein interactions were calculated based on experimental evidence, curated database, and predictions based on gene neighborhood, gene fusion, gene co-occurrence, text mining, co-expression, and protein homology.

## Supplementary Information

Supplementary Information 1.

Supplementary Information 2.

## Data Availability

The mass spectrometry proteomics data were deposited to the ProteomeXchange Consortium via the PRIDE partner repository with the dataset identifier PXD057937.
