# Supplementary material for: The proteome of osteoblasts in a 3D culture perfusion bioreactor model compared with static conditions
Source: Sci Rep. 2025 Apr 9;15:12120. doi: 10.1038/s41598-025-96632-0 (PMC11982442; doi:10.1038/s41598-025-96632-0)
Supplement: Supplementary file 2 — Supplementary Information 2. [file 41598_2025_96632_MOESM2_ESM.pdf]

SUPPLEMENTARY FIGURES:

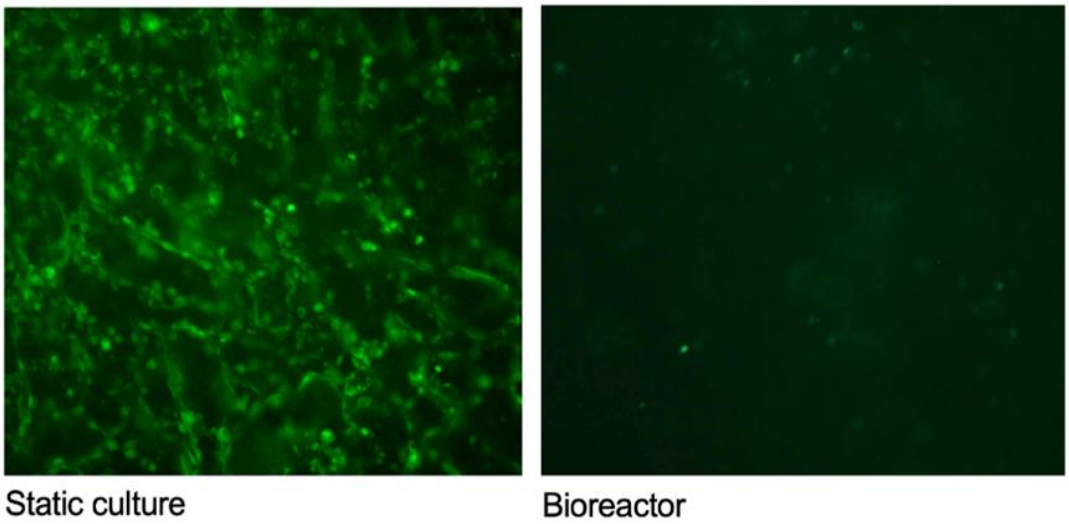

Supplementary figure 1. LIVE/DEAD™ staining. Images of the live cells of the bioreactor and static culture after 21 days is shown.
